# Unprecedented Impacts of Aviation Emissions on Global Environmental and Climate Change Scenario

**DOI:** 10.1007/s40726-021-00206-3

**Published:** 2021-11-10

**Authors:** Farooq Sher, David Raore, Jiří Jaromír Klemeš, Piyya Muhammad Rafi-ul-Shan, Martin Khzouz, Kristina Marintseva, Omid Razmkhah

**Affiliations:** 1 Department of Engineering, School of Science and Technology, Nottingham Trent University, Nottingham, NG11 8NS UK; 2 School of Mechanical, Aerospace and Automotive Engineering, Faculty of Engineering, Environmental and Computing, Coventry University, Coventry, CV1 5FB UK; 3 Sustainable Process Integration Laboratory – SPIL, Faculty of Mechanical Engineering, NETME Centre, Brno University of Technology - VUT Brno, Technická 2896/2, 616 69 Brno, Czech Republic; 4 Cardiff School of Management, Cardiff Metropolitan University, Cardiff, CF5 2YB UK; 5 Department of Systems Engineering, Military Technological College, Al Matar Street, Muscat, 111 Oman

**Keywords:** Environmental pollution, Aviation emissions, Aviation fuels, Air traffic flow, Greenhouse gas (GHG) emissions, Sustainable alternative fuels and COVID-19

## Abstract

There has been a continuously growing trend in international commercial air traffic, with the exception of COVID-19 crises; however, after the recovery, the trend is expected to even sharpen. The consequences of released emissions and by-products in the environment range from human health hazards, low air quality and global warming. This study is aimed to investigate the role of aviation emissions in global warming. For this purpose, data on different variables including global air traffic and growth rate, air traffic in different continents, total global CO_2_ emissions of different airlines, direct and indirect emissions, air traffic in various UK airports and fuel-efficient aircraft was collected from various sources like EU member states, Statista, Eurostat, IATA, CAA and EUROCONTROL. The results indicated that in 2019, commercial airlines carried over 4.5 × 10^9^ passengers on scheduled flights. However, due to the COVID-19 pandemic in 2020, the global number of passengers was reduced to 1.8 × 10^9^, representing around a 60% reduction in air traffic. Germany was the largest contributor to greenhouse gas (GHG) from the EU, releasing 927 kt of emissions in 3 years. In the UK, Heathrow airport had the highest number of passengers in 2019 with over 80 million, and the study of monthly aircraft movement revealed that Heathrow Airport also had the highest number of EU and International flights, while Edinburgh had the domestic flights in 2018. These research findings could be beneficial for airlines, policymakers and governments targeting the reduction of aircraft emissions.

**Graphical abstract:**

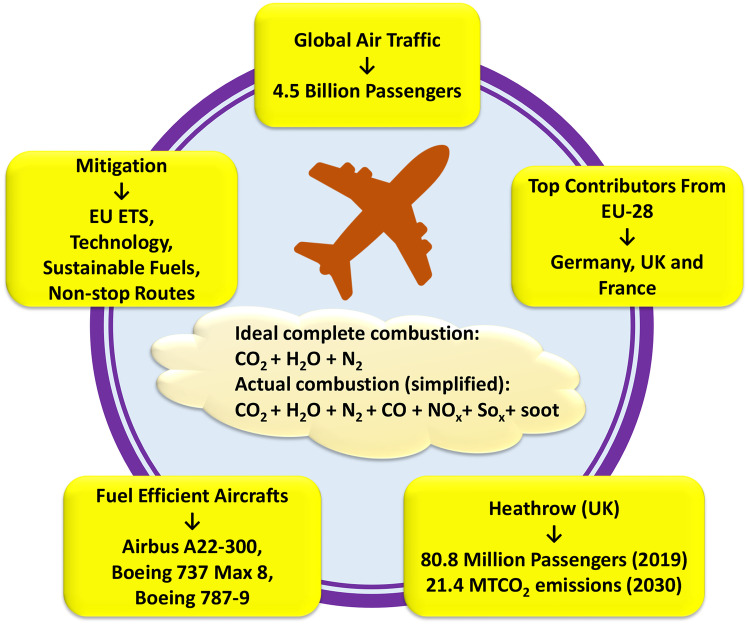

## Introduction

In the last years, every industrial section has shown interest in estimating the negative influence it has on the environment; particularly, the transport industry is the substantial polluter. Air transport occupies a special place due to movement regimes in the high altitude [1]. Civil aviation is primarily derived by globalisation and developing countries, and despite economic crises, the world’s air traffic is rapidly expanding with an average of + 5%/y [2], establishing air transportation as one of the most rapidly growing transport sectors [3]. It is estimated that the daily average oil consumption by global aviation comprises over 5 M barrels (~7.95 × 10^8^ L). The resulting toxic aircrafts’ emissions are a matter of serious concern as they are associated with exposing humans to pollutants that consequently affect their health [4]. Moreover, noise exposure from aircrafts is also linked to an increased risk of health issues such as hypertension [5].

Since the year 1900, the earth’s temperature has increased by 1 °C, while recently, the Arctic has faced enhanced warming of around twice the average rate of global warming. The primary reason for this is the increasing temperature and accumulation of various greenhouse gases (GHGs) [6]. The increase in fossil fuels consumption around the world in the past few decades has enhanced greenhouse gas (GHG) emissions that are leading to climate change [7]. The mitigation of climatic change and decrease in GHG emissions are now among the world’s most important challenges [8]. In the past few years, many studies have used the database of engine exhaust, landing and take-off (LTO) cycle from international civil aviation organisation (ICAO) to evaluate aviation emissions around airports and from aeroplanes [9]. The majority of the gas phase exhaust material from a typical aircraft engine comprises of CO_2_, N_2_, O_2_ and H_2_O. However, various residual products are collectively released in the atmosphere, which includes CO, NO_*x*_, SO_2_ and a huge variety of hydrocarbons. Different aerosol particles are having organic and inorganic components [10].

An aircraft engine can emit about 3.16 kg of CO_2_ and around 1.23 kg of H_2_O for 1 kg of fuel burned [11]. These emissions can further potentially interact with one another, resulting in a higher load on the circumambient atmosphere and on its naturally occurring constituents like carbon dioxide (CO_2_), methane and ozone that can be the indirect impact of warming [12]. Moreover, air pollution caused by aviation resulted in various trace elements such as Pb, Cu, Cd, Cr and Ni is among the most abundant from aircraft emissions [13]. Carbon dioxide (CO_2_) is known as the most abundant carbon-based effluent coming from engines of aircraft and accounts for 72% of total combustion products [14]. Due to the long residing time of CO_2_ in the atmosphere, it plays a major role in climate change from the impact of the aviation sector [15]. The contribution of aviation to the emerging global warming can be estimated alone from CO_2_ emissions, for which the related evidence is present, but the relevant GWP factors (global warming potential) for emissions are deficient [16].

According to a study, the civil aviation industry in China would be responsible for about 0.13 Gt CO_2_ emissions in the year 2020, and between 2020 and 2050, these emissions can increase by 1.6 to 3.9 factor [17]. Nitrogen oxides (NO_*x*_) are also included in the GHG, which are released by aircrafts [18]. NO_2_ has been a matter of concern around many major airports, as high-level concentrations have been found. The UK mean air quality objective of (40 mg/m^3^) has been breached by high (NO_2_) concentrations at Gatwick and Heathrow, London airports [19]. A study conducted on Chania airport Greece estimated the level of NO_2_, and the results at 1 h average concentrations indicated that there were twenty exceedances in the concentration of NO_2_ above 200 μg/m^3^, and two were surpassing the regulated threshold value by the European Union Directive [20]. Sulphur dioxide (SO_2_) in high concentrations has multiple environmental and health effects, and it is also found in aircraft fuel combustion.

A study applied a multipurpose air quality modelling approach to assess air quality at various UK airports and suggested that the strategy of desulphurisation of jet fuel has the potential to greatly reduce human health problems which are associated with aviation. It was also mentioned that some early deaths could be related to UK airport emissions, including SO_*x*_ [21]. Aviation emissions are known to cause 5% of the world’s human-induced radiative forcing, and around 16,000 premature mortalities every year are linked to poor air quality [3]. Particulate matter (PM) released from aircrafts has been associated with various health-related issues, such as cardiovascular, respiratory and lung cancer. Many studies have mentioned that PM, along with other pollutants from aircrafts, has caused problems to global air quality and harmful health impacts to local communities near airports [22]. A study based on the characterisation of PM emissions of an aircraft engine stated that the sizes of emitted particles ranged from 17 to 55 nm and showed a complex morphology. It was also stated that the sampled PM comprised carbon with some traces of calcium, oxygen and sulphur [23].

Currently, extensive research has been conducted close to various airports for a better understanding of ultrafine particles (UFPs) that are produced from aircrafts [24]. Previous assessments of global aviation climate have made different assumptions regarding aviation emissions and aviation operations. The understanding of the effects of aviation on climate has been improved in the past decade but is still incomplete [25], as the challenges linked to the reduction in GHG emissions from the aviation industry are very diverse [26]. In recent years, not enough significant research work has been carried out regarding the contribution of aircraft emissions from the EU and the UK in the increasing global warming trend. For this reason, this study aimed to investigate the role of airlines and airport emissions from the EU and the UK in the current global warming trend, global air traffic, greenhouse gas emissions from the EU and aircraft emissions from different airports in the UK. At the same time, the aircrafts producing the least amount of emissions are also discussed and this study gives a comprehensive view from multiple perspectives regarding the recent situation of aircraft emissions and their contribution to global warming.

## Material and Methods

In order to assess the role of aviation emissions from the EU and UK in global warming, many variables were studied, including the global air traffic, annual growth rate, air traffic in different continents, greenhouse gas emissions total, total global CO_2_ emissions of different airlines and direct and indirect emissions, and air traffic in various UK airports was analysed in detail. For this purpose, data were collected from various resources. The global airline passenger traffic [27], estimated annual growth rate [28] and emissions data [28] were collected from Statista, which is a statistics database that holds data on aviation and IATA (The International Air Transport Association). Information on aviation emissions from EU member states, including greenhouse gas emission statistics [29] and greenhouse gas emissions by source sector [30], was collected from the statistical office of EC (Eurostat).

The record of aviation emissions from the United Kingdom (UK) was analysed with collected quantitative data. For that purpose, the data was sourced from the Civil Aviation Authority (CAA) [31], Eurostat, Atmosfair [32], UK airlines, airports and European Environment Agency (EEA) to study the monthly aircraft movements at Heathrow, Belfast, Cardiff and Edinburgh regarding domestic, EU international and international flights [33]. Information regarding statistics of aircrafts with respect to fuel efficiency was collected from the European Organization for the Safety of Air Navigation (EUROCONTROL) and various articles published online [34], and lastly, the mitigating approaches for aviation environmental impacts were studied from different articles and reviews published online.

## Results and Discussion

### Global Air Traffic and Annual Growth

**Fig. 1:**
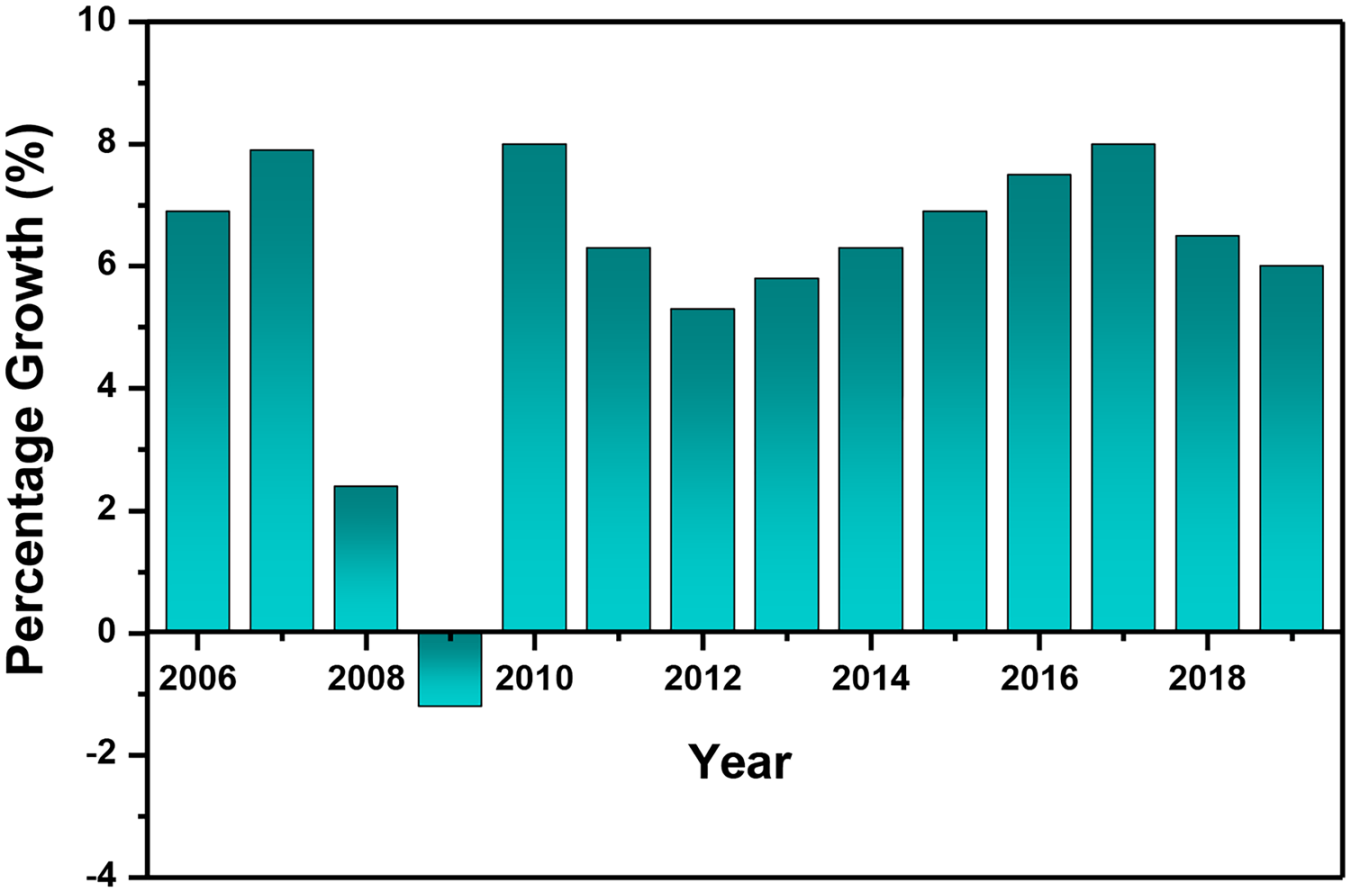
The global annual growth of passenger air traffic from 2006 to 2019

Civil aviation contributes a lot to trade and tourism-related activities in our rapidly globalising world. It has been observed in the last few decades that there is a close link between growth rates of air traffic and the increasing gross domestic product (GDP) of the world [35]. The statistics given in Fig. 1 shows the global annual growth of passenger air traffic between 2006 and 2019. Late 2007 to 2008 shows the recession drop, where passenger demand decreased by 1.2%. In 2010, passenger demand was 8% and then decreased to 6.3% in 2011 and 5.3% in 2012. This decrease was mainly due to economic issues as seen by the EU (European Union) domestic market. The EU domestic market faced regional and national economic decline, with major European airlines reporting large losses, ceasing operations and low passenger bookings [36]. Another percentage of decrease was seen in 2018, as emerging markets faced growing financial market pressures, international trade activity softened, and trade tensions were on the rise [37].

**Fig. 2:**
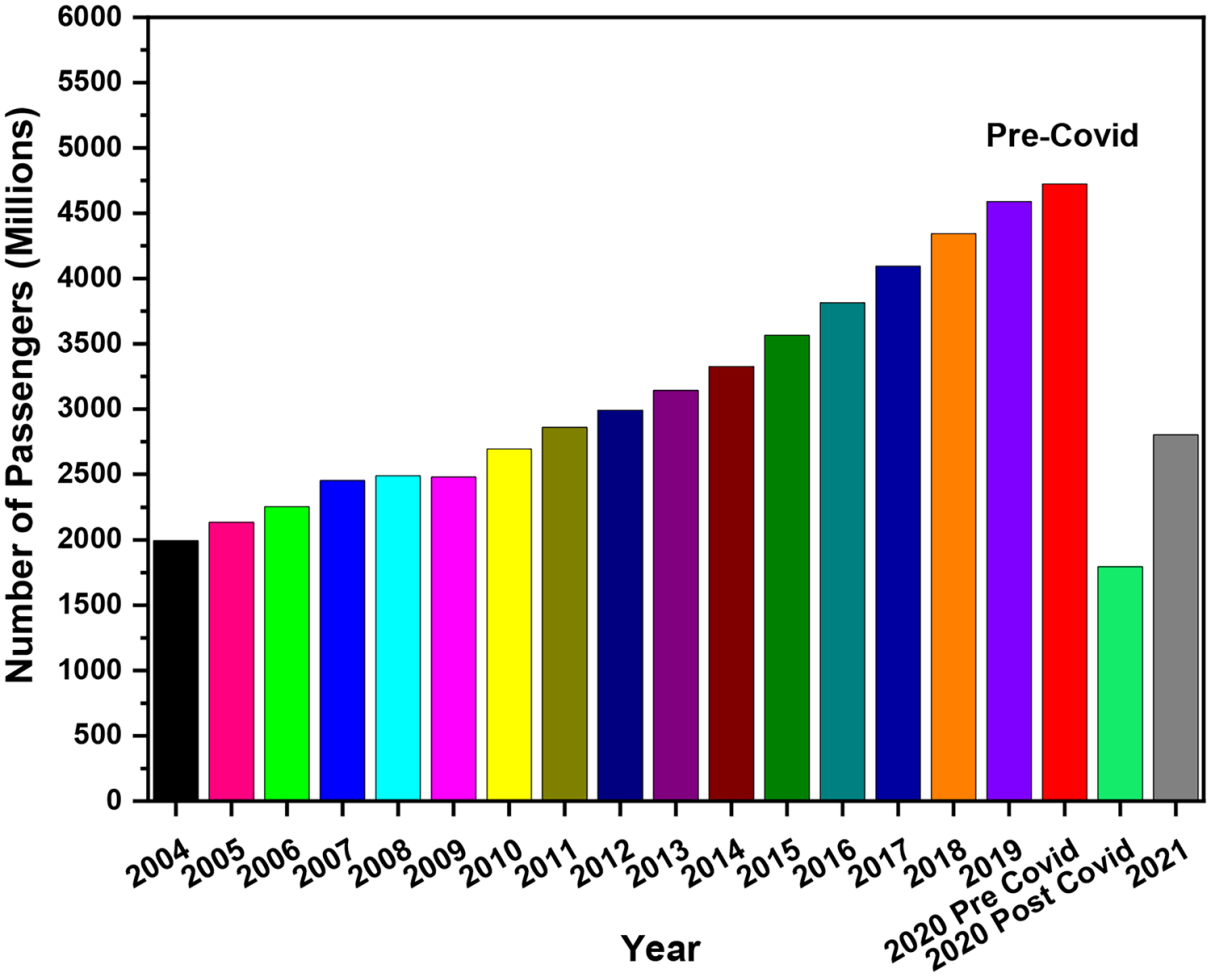
An overview of the total global air passengers boarded aircrafts versus year

The total number of passengers that boarded airline planes from 2004 to 2019 is shown in Fig. 2. Starting from 1.9 × 10^9^ passengers in the year 2004 to 2.1 × 10^9^ in the year 2005, there was a 7.07% passenger increase. This quick increase is possibly due to an increasing amount of people who can afford flights due to increasing incomes. From 2004 to December 2007, which was the beginning of the great recession, there was an increase of 22.9%. However, the great recession brought a slight increase of 1.5% from 2007 to 2008 and then a decrease of − 0.4%. An increase of 8.7% between the years 2009 and 2010 and then increases as the recession started to slow down, leading to a general incline in economic activity. In 2019, commercial airlines carried over 4.5 billion (10^9^) passengers on scheduled flights. Due to the COVID-19 pandemic in 2020, it was seen that the number of passengers boarded by the airline industry around the world was reduced to 1.8 × 10^9^, which represents around a 60% reduction in global air traffic. Movement around the world has been badly affected by the pandemic outbreak. The use of air transportation for travelling purposes has been limited by authorities [38]. The reduction of air traffic is certainly very effective in controlling short-term passenger mobility globally. However, at the same time, it can have high socio-economic impacts [39].

**Fig. 3:**
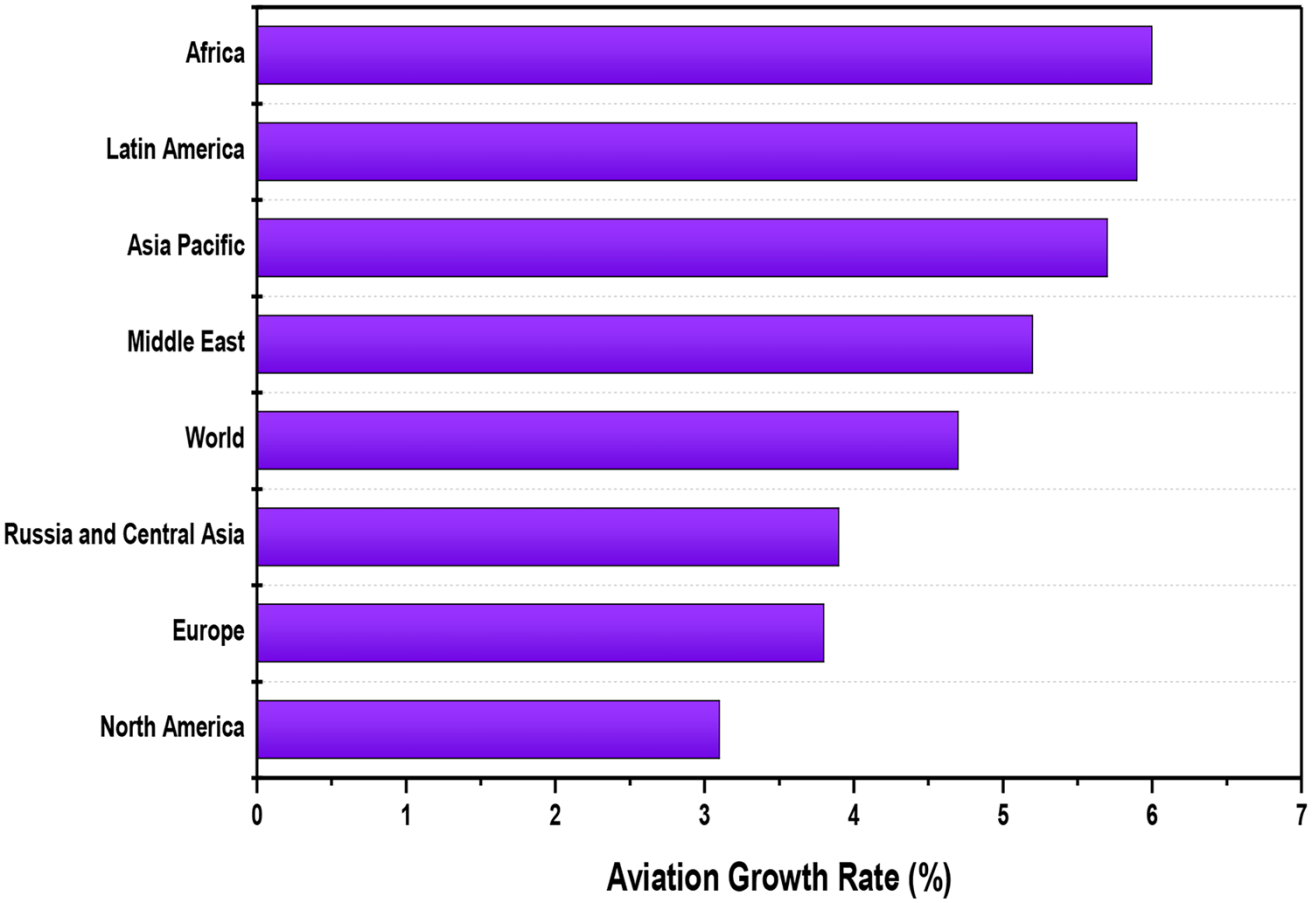
The global annual aviation growth rate in different continents of the world estimated in 2018 and forecasted till 2037

**Fig. 4:**
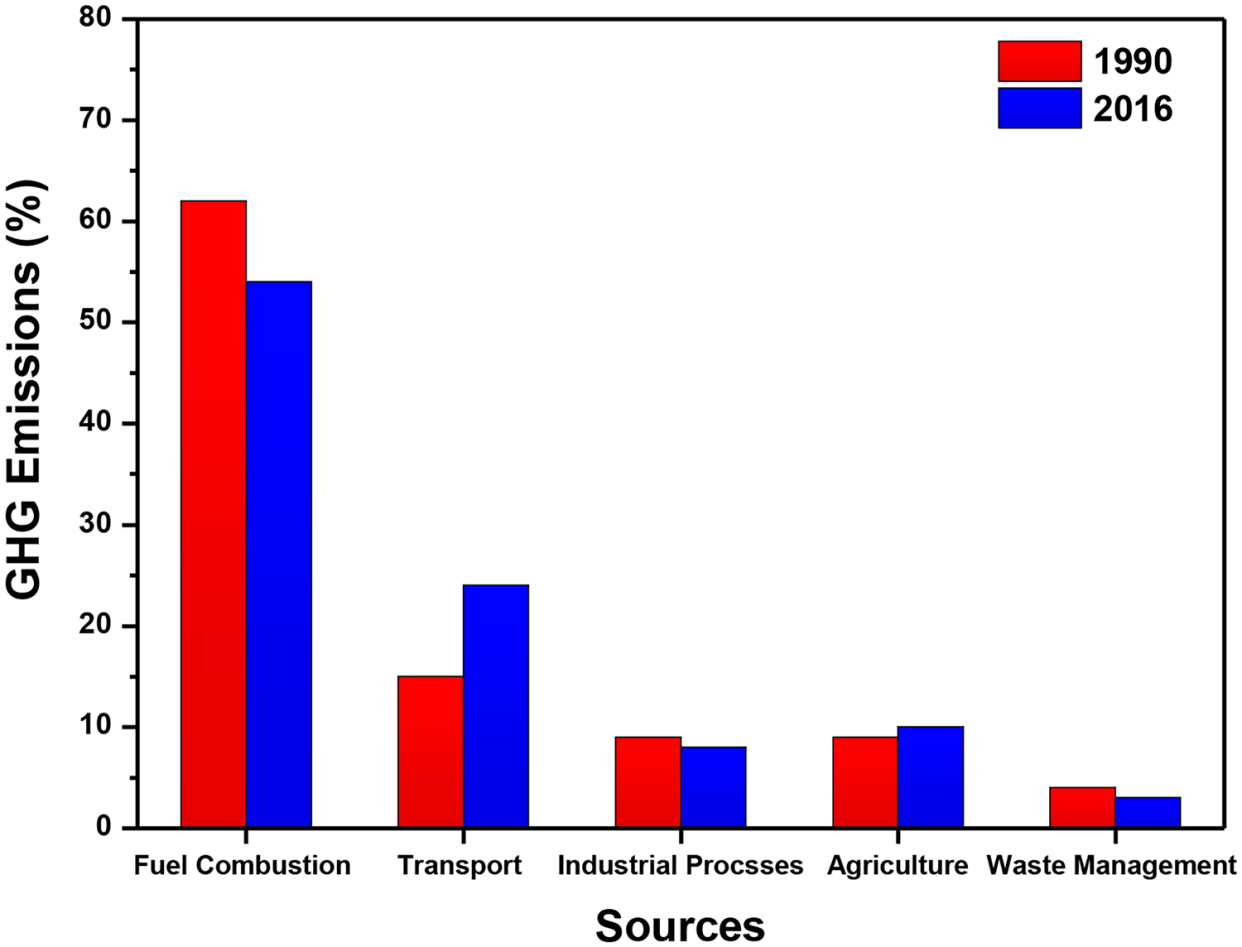
A comparison of various sources of greenhouse gas (GHG) emissions from EU countries in 1990 and 2016

### Air Traffic on Different Continents

The annual passenger air traffic growth rate and cargo air traffic from 2018 to 2037 by continent is given in Fig. 3. By the year 2037, it is estimated that Africa will see a high rate of 6%, followed by Latin America and the Asia Pacific. In the past few years, Africa has achieved a significant level of market growth, and its civil aviation sector has a high growth potential that can benefit this continent greatly. There has been observed an increase in air traffic and aircraft movements, and also in the competition between service quality in those regions in which YD (Yamoussoukro Decision) is implemented [40]. The civil aviation industry in China has undergone huge expansion since the 1980s, and this drastic increase in the aviation sector is expected to be continued [41]. A recent study in Turkey mentioned that in the last 3 years, there had been an increase of around 14.48% in the number of commercial flights, and a 21.14% increase has been observed in the total number of people who travel by air [42].

### Greenhouse Gas Emissions from Aircrafts in the EU

Transport directly affects the quality of daily life of EU citizens in different ways. The total energy consumption from the EU transport sector in 2015 was estimated to be 358.6 Mtoe, which accounts for 33% of the entire EU primary energy consumption, i.e. 1,084 Mtoe [43]. While road transport is the largest segment (82.0%) with a consumption of 293.9 Mtoe, followed by international aviation (12.8%) 45.7 Mtoe, it was estimated that in the European Union, around 973 million passengers would be travelling by air in 2016 with the increment of around 5.9% in comparison of the year 2015 [44]. The greenhouse gas emissions for EU states by their main industries are represented in Fig. 4. In 1990, fugitive emissions and fuel combustion (without transportation) accounted for 62% of EU-28 emissions. In 2016, the figure was 54%, a reduction of 12.9%, which could be due to the use of more fuel-efficient alternatives. On the other hand, transportation (including aviation) accounted for 15% in 1990 and 24% in 2016, a great percentage increase of 60%, making it the second most important source sector.

**Fig. 5:**
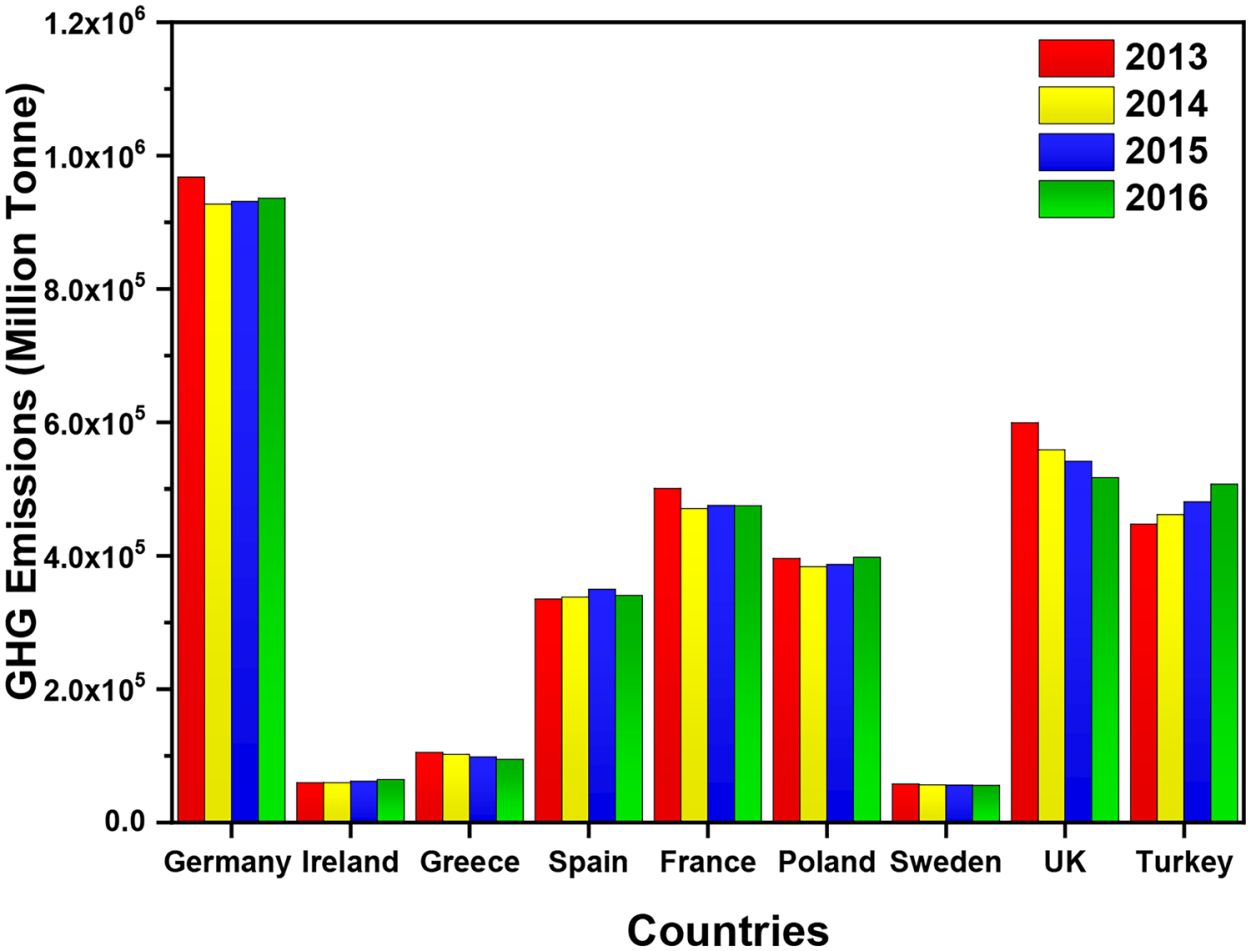
The greenhouse gas emissions (GHG) from nine EU countries from 2013 to 2016. The graph also illustrates the comparison with other countries with respect to GHG emissions

Transport and agriculture both increased in GHG (greenhouse gas emissions), compared to fuel combustion, industrial and waste management industries that saw a decrease between 11 and 25%. Direct emissions from aircrafts and airports account for 2% of global emissions and 3% of the EU total GHG (greenhouse gas) emissions [45]. Greenhouse gas emissions of some of the EU countries were further investigated between the years 2013 and 2016, as shown in Fig. 5. The data represented focused on some of the biggest greenhouse gas emitters, including Germany, the front runner, UK, France, Spain, Poland and Turkey, the latest EU addition. Germany leads on GHG emissions for every year between the years 2013 and 2016, releasing well over 927 Mte greenhouse gas (GHG) emissions, as can be seen in Fig. 5. Germany’s great impact of GHG emissions due to its industrial activities, known for its automotive and manufacturing operations, recorded 967 kt of GHG emissions, which represented a 27% share total in 2013 that was the highest in all the years.

A study concluded that from 16.9 Mt (1995) to 27.3 Mt (2016), aviation emissions in Germany had been increased by almost 60% between 1995 and 2016. However, the study also indicated that in the same time period, the level of specific emissions per kilometre had been declined by around 30% [46]. From 2013 to 2016, there was a 3% decrease in GHG emissions in Germany. Overall, reductions from 2013 to 2016 were also seen by Greece, France, Sweden and the UK. This could be the result of increased internal and external pressures to provide cleaner environments. From the year 2013 to 2014, there was a reduction of GHG emissions across all countries except Spain, which experienced an increase of 0.8% from 335 thousand tonne emissions to 338 Mt. During 2014 to 2015, an increase in emissions was seen by Northern Ireland, Spain, France, Poland and Turkey. The biggest reduction from 2013 to 2016 was seen by the UK with 13%, followed by Greece 9.6%, France 5.2%, Germany 3% and Sweden 2%.

It can also be seen in Fig. 5 that UK comes in second place with the highest GHG emissions, followed by France, Turkey, Poland, Spain, Greece, Sweden and Northern Ireland. The highest number of commercial aircrafts in 2015 was owned by the largest four states of EU and Ireland. A study estimated that the UK had the largest air fleet comprising 1262 aircrafts (19% in EU total), followed by Germany having 1119 aircrafts (17% share) and France with 565 aircrafts (9% share), while Spain had 485 aircrafts (7% share) and Ireland owned 458 (7% share) [47]. It is expected that with the increasing speed of trains, high-speed rail (HSR) can significantly compete with air travel for transportation in Europe. High-speed train lines can potentially substitute medium-haul and short-haul intra-EU flights. HSR services are already available among many EU airports such as Madrid Barajas, Frankfurt Main and Amsterdam Schiphol [48].

### Aircraft Emissions from UK

In this section, airport passenger traffic, greenhouse gas emissions total, direct and indirect emissions were analysed. Data were extracted from various UK airports.

#### Monthly Fluctuation of Air Traffic at Four Major Airports

To take a closer look into the monthly fluctuations of flights in the UK and how they contribute to increasing aircraft emissions, four major UK airports which operate domestic, EU and international flights were investigated. This study gives a closer look at the monthly fluctuations of flights through the year 2018. Edinburgh, Cardiff, Belfast and Heathrow airports were chosen. This data also provides an estimation into the specific months which cause the most emissions. This investigation provides a clearer view of why these airports, such as Heathrow, produce high GHG. Edinburgh recorded its highest number of domestic flights (5,616) in October and lowest in February (4,537). From the statistics collected, it is clear to see that Edinburgh is an airport that focuses more on domestic flights and EU flights than international flights.

**Fig. 6:**
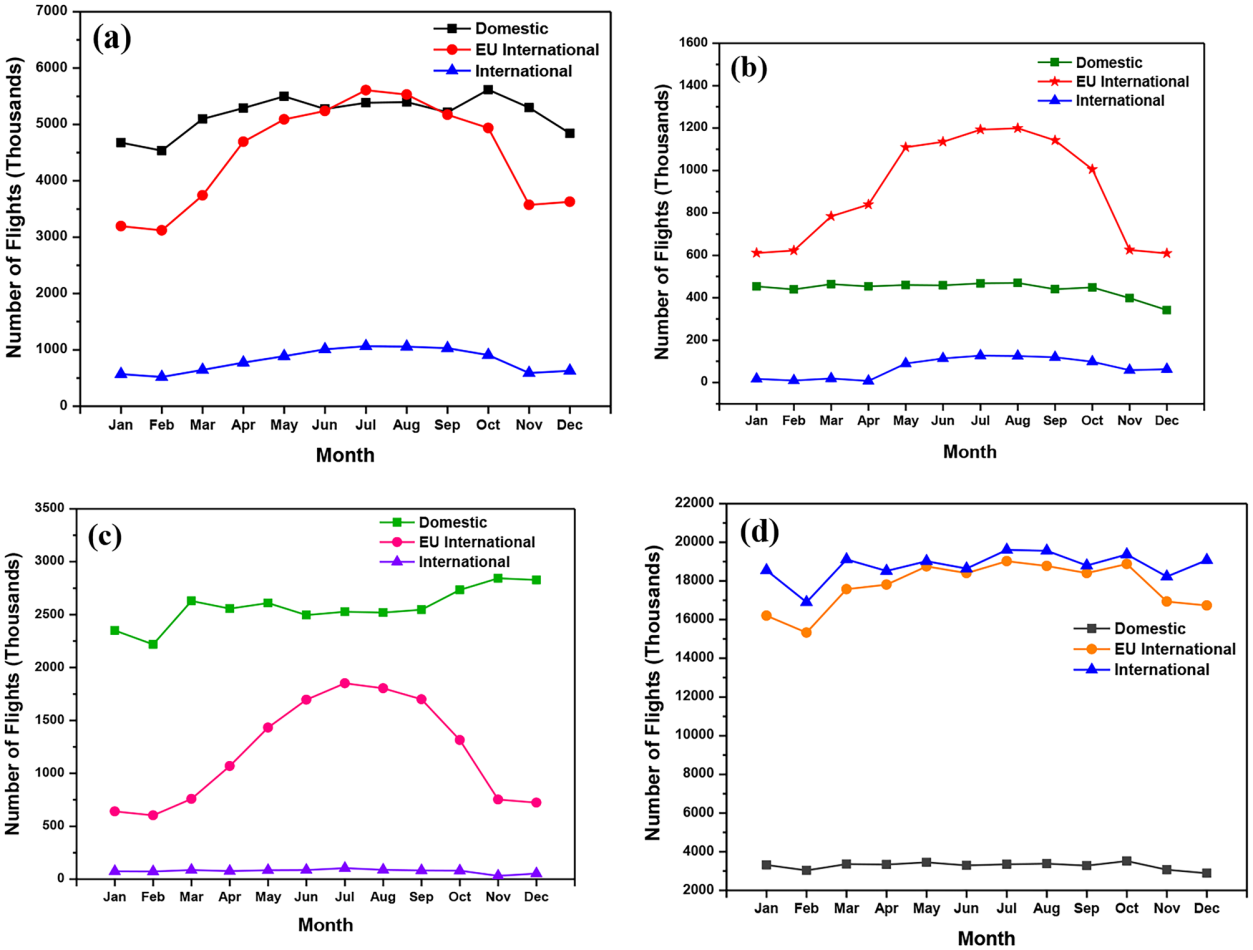
Monthly aircraft movements versus number of flights from the four major UK’s airports; **a** Edinburgh airport, **b** Cardiff airport, **c** Belfast airport and **d** Heathrow airport

Domestic flights were the highest in 10 months out of twelve. January, February and December recorded flights under 5,000 in the category and March to November Flights were over 5,000. Edinburgh seems to experience the highest number of flights between April and October when the weather is mild to warm. In Fig. 6a, domestic flights are low during winter and fluctuate during other months. At the start of the year, EU flights are low and start to increase during summer months, until September, where flights start to decrease, forming an arc shape. This can also be said for international flights, which rise in summer and then decrease in the winter months. Summer months produce the most aircraft emissions due to the high demand for flights. Domestic flights accounted for 50% of the total number of flights, EU 43% and international 7%. According to Edinburgh Tourism, Edinburgh is the 2nd highest city in the UK for international visitors after London. Seventy-seven per cent of tourists are attracted to the historic city and its castles. Thirty-eight per cent of visitors are from outside the UK, and 7% are from the USA, 5% from Germany and 3% from France [49].

Cardiff airport tends to focus on EU international flights. May to October sees an increase in EU international flights and then a decrease into winter. Domestic flights stay constant and remain under 450 flights from January to December. International flights are the lowest in demand, especially in winter months, with April seeing only 8 flights before the sharp increase to 90 flights in May, a 125% increase in Fig. 6b. July and August recorded the highest number of flights for all types of flights. Domestic flights accounted for 31% of flights, EU 64% and international 5%. Domestic flights at Belfast totalled 30,855 flights. Unlike Edinburgh and Cardiff, Belfast recorded its highest domestic flights in November and December. The highest number of EU flights was found in July with 1851 flights, higher than Cardiff’s result of 1,192 but much lower than Edinburgh’s with 5,610. International results remained constant under 105 flights throughout the year in Fig. 6c, suggesting fewer people in Belfast fly to international countries but more to regions and towns closer to home.

**Fig. 7:**
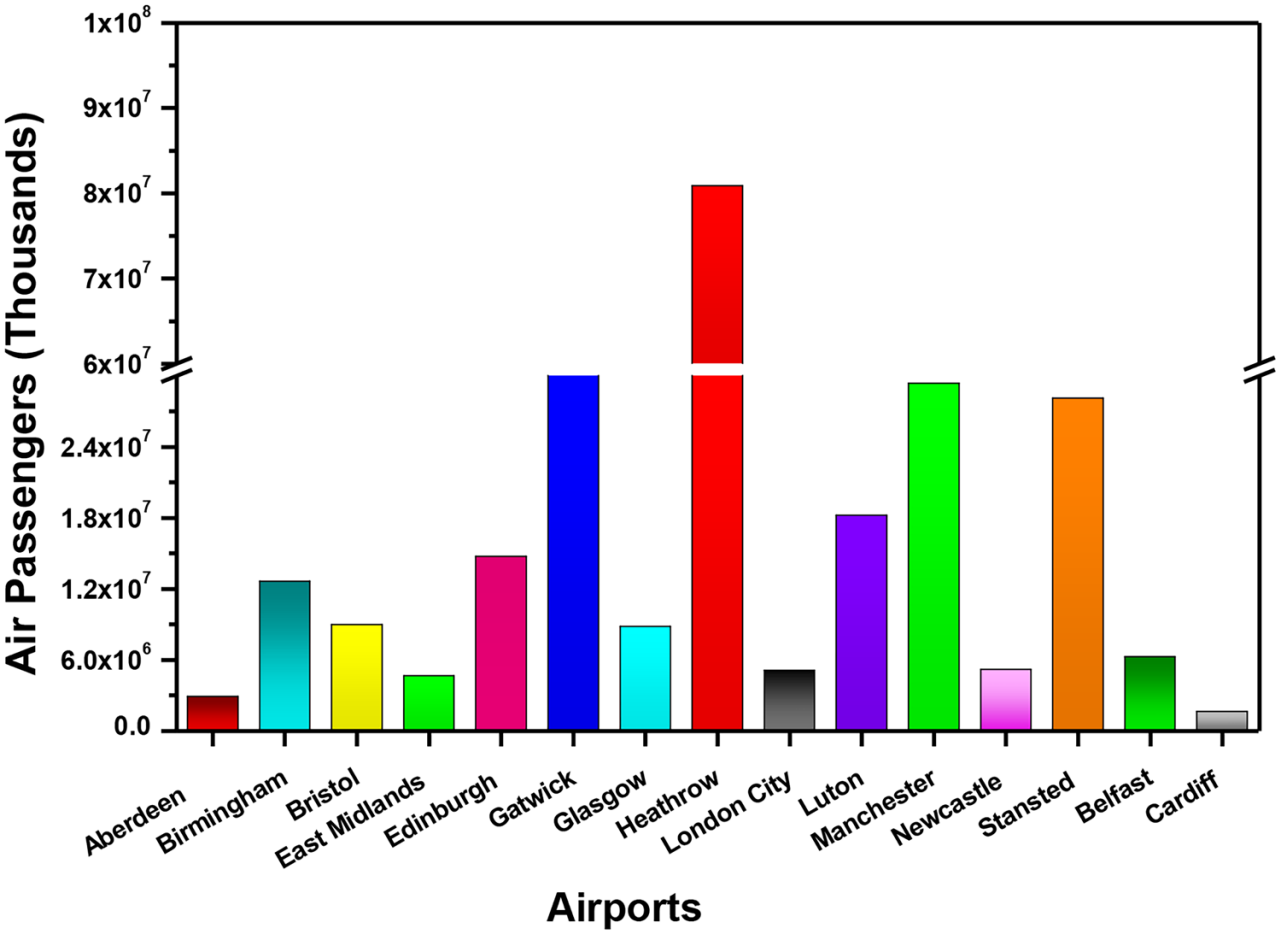
Comparison of total air passengers’ traffic from various airports in the UK

**Fig. 8:**
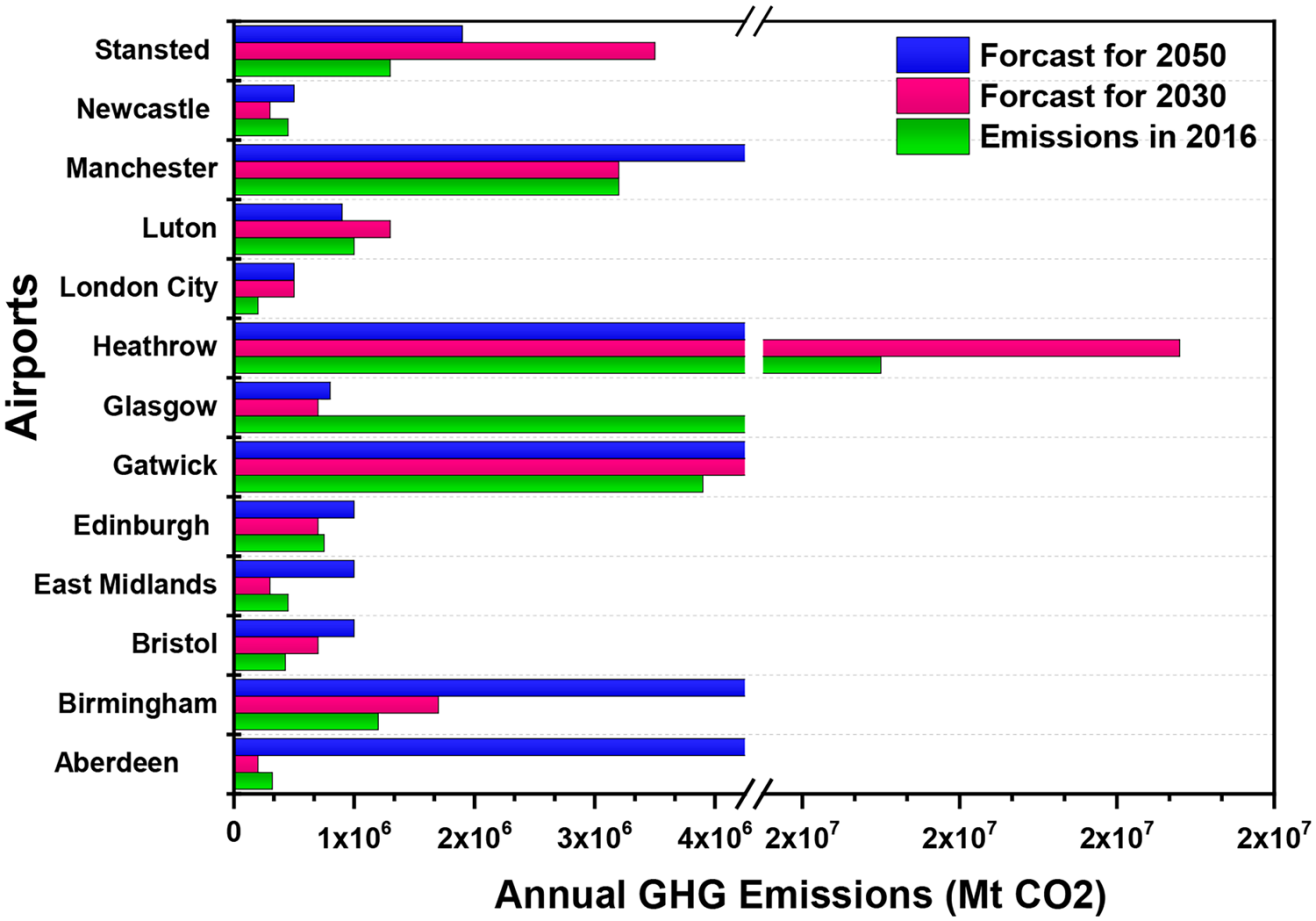
The annual greenhouse gas (GHG) emissions (Mt CO_2_) from thirteen UK’s airports

**Fig. 9:**
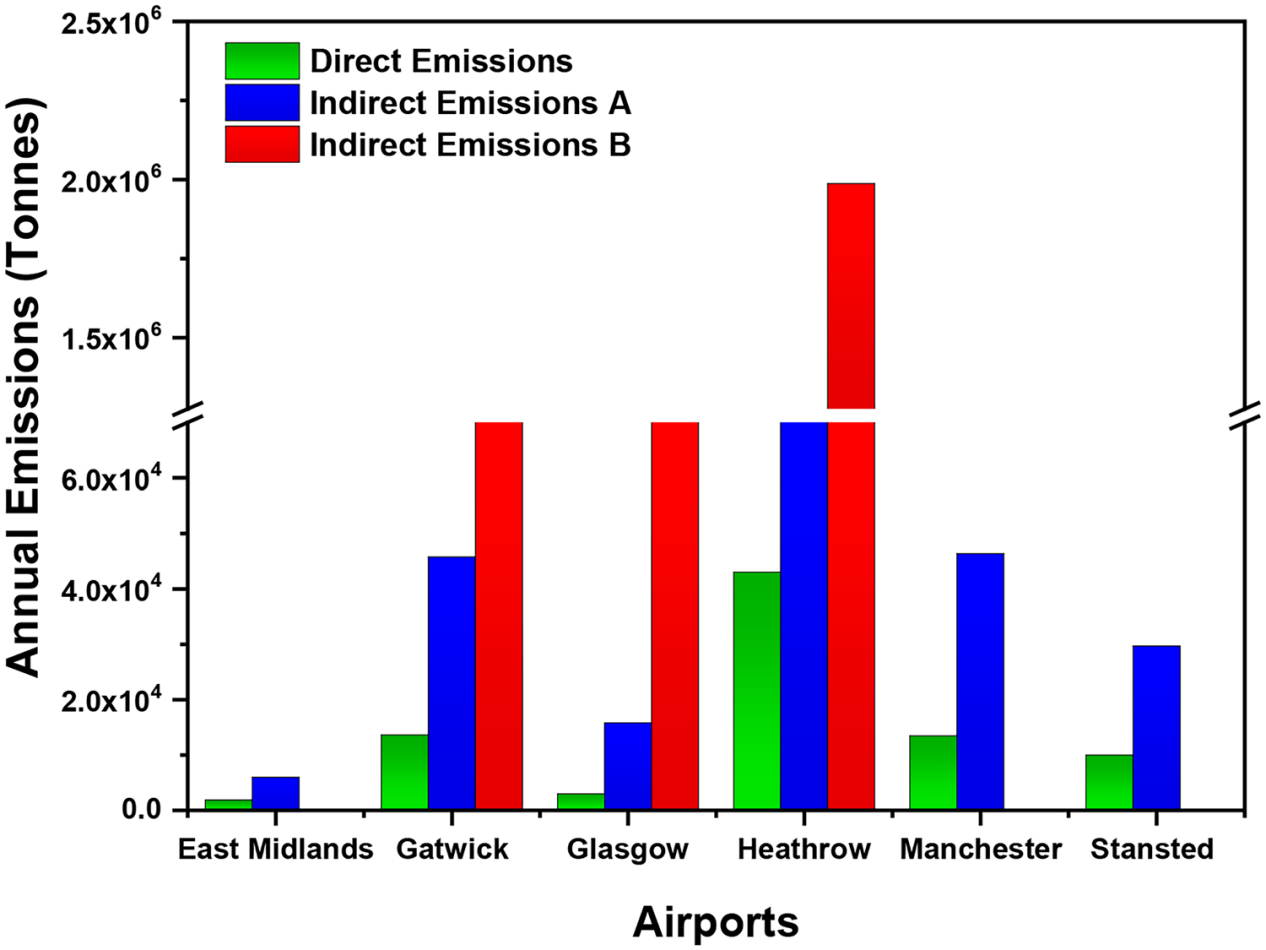
Direct and indirect emissions from the six different airports of the UK. Direct emissions include airport emissions that can be controlled by the airport. Indirect emissions (**A**) comprise airport generated emissions from the buying of heat, steam and electricity. Indirect emissions (**B**) indicate the external emissions that the airport cannot control but are influenced by the airport

However, a high majority of passengers fly to EU countries between April and October. The total number of domestic flights is twice as much as EU international flights. Domestic flights account for 67% of flights from Belfast, EU flights 31% and international 2%. The Heathrow airport of the UK, having international significance, is recognised as a major contributor of pollution and is considered as one of the locations in which the limit values of European air quality have been breached in the past [4]. Figure 6d indicates the monthly fluctuations of flights at Heathrow Airport. It has the highest number of passengers (Fig. 7), GHG emissions (Fig. 8) and indirect and direct emissions (Fig. 9). It has the highest number of domestic flights (39,250) compared to Belfast and Cardiff but not Edinburgh (62,126), a percentage difference of 58%. Heathrow may fail in domestic flights, but it has the highest number of flights in the EU (212,820) and international (225,405) all year round.

Moreover, it is clear to see that Heathrow Airport produces the highest amount of emissions. In research, data collected from 10 sites around Heathrow airport indicated that nitrogen oxides were among the most significant emissions and exceeded the annual mean limit value at various sites [19]. The level of NO_2_ and NO_*x*_ (oxide of nitrogen such as nitrous oxide (N_2_O), nitrogen oxide (NO)) concentrations was estimated based on aircraft emissions, and hourly meteorological data in 3 years following the flight-ban at Heathrow airport (after volcano Eyjafjallajökull eruption in 2010), and the results suggested that the airport closure had a significant effect on NO_2_ and NO_*x*_ concentrations near the airport area, even though the ban had lasted for six days only [50].

#### Air Traffic Flow from Various UK Airports

Heathrow airport has the highest number of passengers in 2019 with over 80 million passengers travelling through the UK’s hub airport as seen in Fig. 7. Gatwick was in second place with over 46 million passengers. In third place was Manchester International Airport, with 29 million passengers followed by London Stanstead with 28 million passengers. Cardiff airport had the lowest number of passengers, under 2 million.

#### Greenhouse Gas Emissions from UK Airports

It is noticeable from Fig. 8 that Heathrow was the top airport in the UK with the highest greenhouse gas emissions. In 2016, Heathrow recorded 19.5 Mt of emissions, a share of 50% of total GHG emissions. The UK’s hub airport is also forecasted to reach 21.4 Mt of emissions in 2030 and then reduce to 18.2 Mt in 2050. Heathrow and Gatwick are both estimated to have an overall reduction in GHG emissions, but Manchester (MIA), who holds a share of 8%, and Stansted with 3% are both forecasted to have an increase in GHG emissions to 5.3 Mt. Aberdeen, with a passenger number of 3.4 million, the second-lowest behind London City airport, is set to see an emission reduction of 37.5% from 320 kt of CO_2_ emissions to 200 kt. London city airports are set to see an emission increase of 150% from 200 to 500 kt. Aberdeen and London City are the only UK Airports set to see no change in emissions forecast for both 2030 and 2050. Bristol and Edinburgh had 428 and 750 kt emissions. However, the same level of emissions forecast for 2030 and 2050 could cause an increase in Bristol emissions by 133% and in Edinburgh by 33%. It can be predicted that Heathrow and Gatwick have both seen reductions in emissions.

Moreover, smaller airports with less number of passengers could influence fluctuations in emissions. Similarly, 6 out of the 14 airports as seen in Fig. 8 are increasing emissions. The smaller airports should have policies in place to reduce emissions. Direct and indirect emissions are shown for 6 airports in Fig. 9. These direct and indirect emissions have been divided into direct emissions, indirect emissions (A) and indirect emissions (B). Direct emissions include airport emissions that can be controlled by the airport. Indirect emissions (A) are composed of generated airport emissions from the buying of heat, steam and electricity. In comparison, indirect emissions (B) include the external emissions that the airport cannot control but are influenced by the airport, such as the aircraft emissions from the landing and take-off cycle and transportation of the passenger to the airport.

As depicted in Fig. 9, Heathrow Airport was the top runner indirect emissions (43,000), indirect emissions A (241,00) and indirect emissions B, a record of 1.9 Mt of emissions, due to having the highest number of passengers of over 80 million and highest number of flights per day of any other UK airport. MIA had the highest indirect emissions generated by the airport with a value of 46,361, compared to the Gatwick value of 45,791 in the same category. East Midlands had the lowest values for direct and indirect emissions. East Midlands, Stansted and MIA did not have indirect emissions B available. Total global CO_2_ emissions of different airlines along with global ranking can be seen in Table 1. United Airlines produced the highest CO_2_ emissions (31.3 Mt) in 2018 and carried 158 million passengers. In second place is Lufthansa which reportedly produced 30.3 Mt CO_2_ emissions in 2017 and carried 142 million passengers in 2018. It has been estimated that since 2009, only one out of five airports in Europe actually participated in the reduction programmes of CO_2_; however, only around 8% have been certified to be CO_2_ neutral [51].

**Table 1:** Total global CO_2_ emissions of different airlines [32]

**Airline**	**Global emissions (Mt)**	**Global passengers carried (M)**	**Efficiency points (EP)**	**Global ranking (2018)**
**Aer Lingus**	N/A	10.4 (2016)	N/A	N/A
**American Airlines**	26.8 (2011)	203 (2018)	58.7 (2018)	58
**British Airways**	18.1 (CO_2_)	45 (2018)	54.4 (2016)	74
**EasyJet**	7.6 (2018)	88.5 (2018)	N/A	N/A
**Emirates**	25.6 (2017)	58.5 (2018)	40.7 (2016)	108
**Flybe Ltd**	N/A	9.5 (2018)	40.8 (2016)	98
**Jet2**	N/A	9.6 (2017)	0.70.8 (2018)	11
**Lufthansa**	30.3 (2017)	142 (2018)	56.9 (2016)	66
**Ryanair**	N/A	139.2 (2018)	N/A	N/A
**Thomas Cook**	6.8 (2017)	20 (2018)	78.6 (2018)	7
**Tui Airways**	66.7 g CO_2_ (2017)	27 (2018)	79.2 (2018)	1
**United Airlines**	31.3 (2018)	158 (2018)	60.4 (2018)	50
**Virgin Atlantic**	N/A	5.4 (2018)	N/A	83
**Air Canada**	N/A	44.8 (2018)	65.6 (2018)	32
**Air France**	N/A	49.8 (2018)	54.5 (2016)	73

In 2014, Lufthansa flew a commercial flight with 10% of Farnesane, a biofuel component. In 2011, Lufthansa was the first airline in the world to use biosynthetic fuel on its European scheduled flights [52]. Before Thomson Airways turned into TUI airways, it carried 230 passengers on a flight to Lanzarote on one engine powered by hydro processed esters (biofuel) and another engine with regular jet fuel back in 2011 [53]. British Airways and Virgin Atlantic are currently working on household waste and carbon gas. It can also be seen in Table 1 that Tui Airlines, the British holiday airline, was ranked first in the 2018 global efficiency chart, due to its low carbon emissions per passenger of 66.7 g per revenue passenger kilometres and reaching under 80% of optimum level carbon emissions, with its 79.2 efficiency points value. However, its British counterpart, Flybe (now not operating anymore), received an efficiency point of 40.8 in 2016 and ranked in 98th position. Out of all airlines that operate in and out of the UK, the Emirates was the lowest-rated with a global efficiency position of 108 and an efficiency point value of 40.7, putting it at the lower end of the spectrum.

### Fuel-Efficient Aircrafts

The amount of CO_2_ emissions released from a flight depend on various factors. Airlines, airports and regulators can control some of the factors, but some cannot be controlled, such as weather. One very important factor which contributes to aviation emissions is the aircraft type. The aviation market is composed of a very limited number of producers that are in high competition with one another. Technology is regarded as the most basic parameter in this sector, and currently, the entire focus is on energy efficiency [44]. From the middle of the previous decade, new aircraft types like the Airbus A320neo, Airbus A350 and Boeing 787 were introduced, which have about 15% improvement in CO_2_ emissions per revenue tkm as compared to the predecessors [46]. Table 2 enlists the aircrafts categorised by short, medium and long-haul travel. These are the most fuel-efficient planes currently used by airlines that operate in the UK and outside.

**Table 2:** Types of aircrafts used for different flights including short, medium and long haul along with their total number of seats, travelling distance, load factor, fuel burn and fuel per seat ratio

**Flight type**	**Type of aircraft**	**Number of seats**	**Travelling distance**	**Load factor**	**Fuel burn**	**Fuel per seat**	**Reference**
			**(km)**	**(%)**	**(kg/km)**	**(L/100 km)**	
Short haul	Airbus A319 Neo	136	6,850	68	2.40	1.93	[86]
	Boeing 737 Max-7	140	7,130	65	2.51	1.94	[86]
	Airbus A220-300	135	5,920	72	2.30	1.85	[87]
Medium haul	Boeing 787–8	291	6,300	75	5.26	2.26	[88]
	Irkut MC-21	163	3,240	73	3.04	2.33	[89]
	Boeing 787–9	304	6,200	75	5.77	2.37	[90]
Long haul	Boeing 787–9	304	9,208	75	5.63	2.31	[90]
	A350-900	315	9,208	80	6.03	2.39	
	Airbus A330neo-900	300	8,610	76	5.94	2.48	[91]
	Airbus A330neo-800	248	8,610	74	5.45	2.75	[91]

In the short-haul category, Airbus A220-300 has the lowest fuel burn of 2.30 kg/km and fuel per seat of 1.85 L/100 km. Lufthansa and Delta, known for being high CO_2_ emitters, in March 2018 ordered 20 and 50 Airbus A220-300 planes. Currently, no British airlines are operating this aircraft [54], while A319Neo is the second most efficient aircraft in the short-haul category. In medium-haul flights, the most efficient plane is Boeing 787–8 in terms of fuels per seat, while, with respect to fuel burn, Irkut MC-21 is the most fuel-efficient. The most fuel-efficient long-haul aircraft is the Boeing 787–9 due to its fuel economy of 2.31 L/100 km; however, it has a fuel burn 5.63 kg/km. It is usually assumed that larger aircrafts are more fuel-efficient per passenger due to the economies of scale. However, this is not the case as larger aircrafts burn more fuel and release more carbon into the atmosphere compared to their smaller peers [55].

As compared to medium- and long-haul flights, the emissions of short-haul flights are much lower. However, short-haul flights are responsible for producing an increased amount of emissions per tkm. This can not only be because the energy-intense climb and take-off phase is dispensed over the much shorter distance of flight but also because of decreased load factors and the less amount of cargo that is carried in comparison to long- and medium-haul flights. A study conducted in Germany concluded that short-haul flights produced twice as high CO_2_ emissions (1653 kg) as compared to emissions from long-haul flights (706 kg/tkm) [46]. The average fuel consumption on each route is influenced by the different mix of a small regional jet (SJ) and narrow-body (NB) aircraft, which depends on the available fleet of the airlines performing the service [44].

### Mitigating Approaches for Aviation Environmental Impacts

Concerns have arisen with the ongoing environmental impacts of aviation; control strategies to reduce the devastating effects have become the priorities for the aviation industry [56]. However, the global nature of the aviation sector has made it more difficult for new strategies to be implemented. The air traffic is expected to increase at 4.5% rate/year in the coming few decades, and there are serious concerns for the aviation sector regarding the emissions and the accessibility of various fuel resources for future use [57]. Aircraft emission reduction demands a combination of technological, market-based operational and economic measures to be followed through, with a set of short, medium and long-term goals [58]. Below are some recommended measures suggested in the light of already published literature and research for reduction of the aircraft emissions worldwide.

#### Emissions Trading System for the Reduction of EU GHG Emissions

The European Union emissions trading system (EU ETS) was set up in 2005 and is the world’s first major international emissions trading system and the biggest one. It is set out in Article 17 of the Kyoto Protocol [59]. The EU ETS is a key tool for reducing EU greenhouse gas emissions cost-effectively. This tool uses the cap and trade principle. It can allow the companies to invest in low-carbon clean technologies, and it covers 45% of released carbon emissions from EU greenhouse gas emissions. According to the EU ETS, the year 2020 might be having a 21% reduction in emissions as compared to 2005. Under this current system, 2030 should see a reduction of 43% [60]. Mitigation strategies like the EU ETS are essential to change behaviour towards air travel and also to incorporate technological and operational changes in the aviation sector, resulting in decreasing environmental impacts [61].

#### Technological Improvements

Sustainable aviation can be reached through investments in technological improvements, along with other measures [62]. Present modern aircrafts produce 80% less CO_2_ per seat than the first jets designed in 1950. However, there is still room for improvement [63]. In March 2017, the International Civil Aviation Organisation (ICAO) adopted a new CO_2_ emissions standard for aircraft, which applies to all new aircraft type designs from 2020 and new aircraft type designs currently in production as of 2023 [64]. Wingtip devices that are currently installed by manufacturers reduce fuel usage and increase overall aerodynamic efficiency [65].

#### Sustainable Alternative Fuels

In the past few years, many industrial initiatives have gained significance to search for alternative ways of attaining biofuels for aviation; that is why there has been increasing research regarding alternative fuels composed of biomass [66]. Substituting fossil-based jet fuels with biomass-based jet fuels can help in reducing aviation emissions and energy crises [67]. Table 3 enlists the stage of biofuel development by different airlines, along with the proposed sources. Many airlines and manufacturers have invested heavily to find sustainable alternative jet fuel to reduce greenhouse gas emissions of the air transport industry. Manufacturers such as Boeing are currently in the production of aircraft that can transport passengers solely on sustainable alternative fuels [66]. Biofuels should not be sourced from valuable resources, competing with food sources, but sustainable ones, and they can be produced in many geographical locations.

**Table 3:** The stage of biofuel development by different airlines along with the proposed sources

**Airline**	**Biofuel development commitment**	**Proposed source**	**Source**	**Reference**
British Airways	In place	Converting household waste to renewable jet fuel	British Airways Sustainable Fuel report	[92]
Lufthansa	In place	Tested out a biokerosene mixture	Lufthansa Balance sustainability report	[93]
Thomson (TUI)	Was in place	Use of Hydro processed esters and fatty acids (HEFA) fuel- cooking oil	Reuters	[94]
Virgin Atlantic	Testing waste gases	October 2018- Flight using waste carbon gas from a steel mill	Virgin Atlantic Press Release	[95]

The second-generation biofuels can compete with stable prices of the current jet kerosene fuel [68]. The Australian aviation industry is planning to supply 5% of the domestic fuel from biomass by 2020 [69]. However, biofuels can produce high-level NO_*x*_ emissions [70]. Other than biomass, other raw materials are also available, which can be used in the production of sustainable fuels for aircrafts. HEFA (hydrotreated esters and fatty acids) and Fischer–Tropsch jet fuels are considered the most promising synthetic jet fuels as their large-scaled plants are already in existence [71]. Recently, it has been reported that scientists are now able to convert CO_2_ into jet fuel with the help of a cutting-edge method bypassing it from an iron catalyst with hydrogen, manganese, potassium, and citric acid. Additionally, this method is claimed to be less expensive than other means of jet fuel production [72].

#### Role of Electric Aircrafts

As the aviation sector is progressing towards a greener approach [73], one way to achieve zero emissions is to use electric aircrafts [74]. In comparison to conventional aircrafts, electric aircraft (MEA) that are less dependent on fuels that are carbon-based eventually produce less NO_*x*_ and carbon emissions. Moreover, they have more reliability and efficiency and produce less noise that could result in ending the ban on night-time flights at various airports [75]. While the existing battery technology may not support long- and medium-haul flights at a full capacity, the First Generation Electric Aircraft (FGEA) can significantly contribute to short-haul flights in the future [74]. MagniX, an electric motor manufacturing company for aircrafts, conducted a grand caravan with the world’s largest electric aircraft in May 2020, along with AeroTEC. Conducting a 1.5-h flight with Cessna Grand Caravan had a cost of power of US$24.68 only compared to conventional fuel, whose cost was around US$404.55 [76]. The MagniX is now also working to provide 300 aircraft fleet to the UK by 2030 [77]. The accelerating development of electric cars also brought intensive innovations to develop cheaper, and for avionic industry important, lighter batteries. This offers important benefits for the future development of electric planes.

#### The Use of Non-Stop Routes

Air traffic management (ATM) is a programme to follow the best fuel-efficient and environmentally friendly routes. It has a significantly important role in decreasing the impacts of aviation on the environment, which is achieved by reducing the inefficient activities in the routes flown by aircraft [78], and non-stop routes to destinations could reduce the level of carbon emissions aircrafts. The emissions of carbon for both connecting and direct routes between ten heavily populated areas in the USA, along with thirteen different tourist destinations situated in the Western and Sunbelt regions in the USA, were studied. It was seen that direct routes had generally outperformed the connecting routes relating the carbon emissions with several exceptions. Furthermore, it was seen that, on average, non-stop pathways decreased carbon emissions roughly by 100 kg/person as compared to the adjoining best option of flight [79]. Another study concluded that for short trips (< 500 km), aviation is not a fuel-efficient option mainly because of large emissions associated with landing and take-off and also due to emissions from ground support equipment related to any trip [80].

## Impact of COVID-19 on Global Aviation

In March 2020, the European Union (EU), for the first time, closed all external borders in order to control the spread of COVID-19, following various governments implementing massive travel restrictions and border control to mitigate the outbreak of this global pandemic [81], which affected air transport and airline industry all over the world [82]. A study indicated that in the EU, COVID‐19 gradually affected air transport, and a peak reduction of 89% in the total number of flights was observed in April [82]. However, the restrictions imposed due to the pandemic has led to improved air quality around the globe. A study conducted in the USA compared historical data (pre-COVID-19) with current periods of COVID-19 to observe particulate matter (PM 2.5) and NO_2_ concentration. The results suggested that the concentrations of both the pollutants have significantly reduced in the atmosphere during the COVID-19 pandemic [83]. Similarly, reductions in PM 10, SO_2_, CO, O_3_ and NH_3_ are also reported in India in comparison to the pre-COVID-19 period. Moreover, there has been around 11.4% improvement in China’s air quality during the pandemic in comparison to the pre-COVID-19 period [84]. A good aviation system must also evaluate the risks, and the cost of such risks is included in the price that is paid by the consumers. Therefore, during the COVID-19 pandemic, there is an opportunity for the air transport industry to increase their profitability and also to re-examine air transport around the world [85].

## Conclusion

Aviation emissions are a significant contributor to Global warming. Global aviation will inevitably continue to expand due to the increase in the global population. The number of passengers increased from 1.9 × 10^9^ in 2004 to 2.1 × 10^9^ in 2005, resulting in an overall 7.07% increase in global air traffic. Developing countries with high population densities have seen high levels of pollution. From the EU, Germany was the highest producer of GHG emissions, releasing well over 927 Mt emissions, followed by the UK and France in the second and third place respectively. In 2019, commercial airlines carried over 4.5 × 10^9^ passengers on scheduled flights; however, in 2020, due to the COVID-19 pandemic, the number of passengers around the world were reduced to 1.8 × 10^9^ (around 60% reduction in global air traffic). In the UK, Heathrow Airport had the highest number of passengers in 2019 (over 80 million) and Gatwick was in second place with over 46 million passengers. Among the four UK airports, it was identified that Heathrow Airport has the highest number of EU and International flights. However, Edinburgh has the highest domestic flights in the UK. The data shows that United Airlines and Lufthansa are the highest contributors to CO_2_ emissions compared with other airlines, depending on short-, medium- and long-haul flights as an example of fuel-efficient aircrafts can serve, e.g. Airbus A22-300, Boeing 787–8 and Boeing 787–9. It is important to adopt suitable measures to control and decrease the impact of aviation emissions on increasing global warming, which can be achieved through collective cooperation between states and organisations that require major changes to current Government policies. This study has recommended technological advancements, emissions trading such as the European Union Emissions Trading System (EU ETS), the use of sustainable alternative fuels, electric aircrafts and the use of non-stop routes.

## Data Availability

Not applicable.
